# Lipoprotein particles and coronary artery calcium in middle-aged US-White and Japanese men

**DOI:** 10.1136/openhrt-2019-001119

**Published:** 2019-12-29

**Authors:** Hemant Mahajan, Maryam Zaid, Rachel Mackey, Aya Kadota, Abhishek Vishnu, Akira Fujiyoshi, Ahuja Vasudha, Takashi Hisamatsu, Rhobert Evans, Tomonori Okamura, Katsuyuki Miura, Lewis Kuller, Hirotsugu Ueshima, Akira Sekikawa

**Affiliations:** 1 Graduate School of Public Health, University of Pittsburgh, Pittsburgh, Pennsylvania, USA; 2 Shiga University of Medical Science, Otsu, Shiga, Japan; 3 Shimane University Faculty of Medicine Graduate School of Medicine, Izumo, Shimane, Japan; 4 School of Medicine, Keio University, Tokyo, Japan

**Keywords:** atherosclerosis, lipoproteins, coronary artery disease

## Abstract

**Objective:**

This cross-sectional study examined whether contrasting distributions of nuclear magnetic resonance (NMR)-measured lipoproteins contribute to differences in the prevalence of subclinical atherosclerosis measured using coronary artery calcium (CAC) between the two groups of middle-aged males: the US-residing Caucasian (US-White) and Japan-residing Japanese (Japanese).

**Methods:**

In a population-based study of 570 randomly selected asymptomatic men aged 40–49 years (270 US-White and 300 Japanese), we examined the relationship between race/ethnicity, NMR-measured lipoproteins and CAC (measured by Electron Beam CT and quantified using the Agatston method) using multivariable robust Poisson regression adjusting for traditional and novel risk factors for coronary heart disease (CHD).

**Results:**

The US-White compared with the Japanese had significantly different NMR-measured lipoprotein particle distributions. The US-White had a significantly higher prevalence of CAC≥10 (CAC-prevalence) compared with the Japanese adjusting for CHD risk factors (prevalence ratio (PR)=2.10; 95% CI=1.24 to 3.48), and this difference was partially attenuated (~18%) with further adjustment for lipoprotein levels (PR=1.73; 95% CI=1.02 to 3.08). There was no reclassification improvement with further addition of lipoproteins particle concentrations/size to a model that already included traditionally measured lipids (low-density lipoprotein cholesterol, high-density lipoprotein cholesterol, and triglycerides), cardiovascular risk factors, and inflammatory markers (net reclassification improvement index=−2% to 3%).

**Conclusions:**

Variations in the distribution of NMR-measured lipoprotein particles partially accounted for the difference in the CAC-prevalence between middle-aged US-White and Japanese men.

**Key messages**

**What is already known about this subject?**

- We previously have reported a much higher prevalence of subclinical atherosclerosis measured by coronary artery calcium (CAC) in middle-aged US-residing Caucasian men (US-White) than Japanese men residing in Japan (Japanese) despite Japanese have higher rates of smoking and hypertension, and similar levels of serum total cholesterol, low-density lipoprotein cholesterol, and diabetes.
- Differences in the prevalence of CAC between the two race/ethnicities cannot be attributed to lifetime exposure to traditional risk factors, different genetic make-up or genetic responses to various risk factors.

**What does this study add?**

- The asymptomatic middle-aged US-White men compared with the Japanese men had significantly different lipoprotein particle distributions: the middle-aged US-White men had higher concentrations of small high-density lipoprotein particles (HDL-P), large very low-density lipoprotein particles (VLDL-P); lower concentrations of intermediate-density lipoprotein particles, total HDL-P, medium HDL-P, large HDL-P, small VLDL-P; larger VLDL particle size and smaller HDL particle size.
- The US-White men had 2.10 times higher prevalence of CCS≥10 (CAC-prevalence) compared with the Japanese adjusting for traditional cardiovascular risk factors, alcohol consumption, and inflammatory markers.
- This higher prevalence of CCS≥10 in the US-White was partially attenuated with further adjustment for large HDL-P and large VLDL-P/VLDL particle size.

**Key messages**

**How might this impact on clinical practice?**

- This study highlights that the differences in the distribution of nuclear magnetic resonance-measured lipoproteins between US-White and Japanese men *partially* accounted for the differences in CAC-prevalence between two populations independent of traditional and novel cardiovascular risk factors.
- Our findings support the notion that there is a common source exposure in the diet among the Japanese, which may account for their lower rates of atherosclerosis and coronary heart disease.

## Introduction

Coronary artery calcium (CAC), a well-established biomarker of coronary atherosclerosis—the major underlying cause of coronary heart disease (CHD), has a strong and graded association with atheromatous burden found in the coronary arteries.^1^ Both baseline CAC score^1^ and its progression^2^ predict future CHD among men and women of all ages and of various ethnicities. Cardiovascular disease (CVD) mortality^3^ studies and autopsy studies of atherosclerosis^4^ have reported a much higher burden of coronary atherosclerosis among US-residing white men (US-White) compared with Japanese men residing in Japan (Japanese). Furthermore, we have reported a much higher prevalence of CAC (CAC-prevalence was defined as Agatston’s CAC score ≥10) among US-White compared with Japanese despite the fact that Japanese have had higher rates of smoking and hypertension, and similar levels of serum total cholesterol (TC), low-density lipoprotein cholesterol (LDL-C) and diabetes.^5^

The enzymatically measured lipid concentrations (traditional lipids): LDL-C, high-density lipoprotein cholesterol (HDL-C) and triglycerides (well-established risk factors for CHD^6^ are not virtually equal to lipoprotein particle concentrations (low-density lipoprotein particles (LDL-P), high-density lipoprotein particles (HDL-P) and very low-density lipoprotein particles (VLDL-P)) measured by nuclear magnetic resonance (NMR) spectroscopy; residual risk of CHD remains even after achieving recommended levels of LDL-C and HDL-C with medications. The inability to eliminate CHD risk completely by normalising LDL-C and HDL-C led researchers to shift focus to NMR-measured lipoprotein particle concentrations, which have been suggested as alternative biomarkers for improved risk assessment of atherosclerosis and CHD.^7–9^ Studies have reported that NMR-measured small LDL-P, large LDL-P, total LDL-P, large VLDL-P, total VLDL-P and total HDL-P are significantly associated with subclinical atherosclerosis and CHD/CVD.^9–15^ In fact, some studies have shown that NMR-measured lipoprotein particle numbers (total LDL-P and total HDL-P) are independent and more robust predictors of atherosclerosis and CHD/CVD events than their cholesterol counterparts (LDL-C and HDL-C).^12–15^

We have previously reported differential distributions of NMR-measured lipoproteins between US-White and Japanese.^16^ To the best of our knowledge, however, no previous study has examined whether these results (differences in the distributions of NMR-measured lipoproteins) account for the difference in the CAC-prevalence between US-White and Japanese. Therefore, we aimed first, to evaluate the association between NMR-measured lipoproteins and CAC in asymptomatic US-White and Japanese men aged 40–49 years, and second, to assess the role of NMR-measured lipoproteins in determining the difference in CAC-prevalence between the two populations using the ERA-JUMP study data (the electron beam computed tomography (EBCT), risk factor assessment among Japanese and US men in the post-World War II birth cohort).

## Materials and methods

### Study population

We have previously described the details of the study protocol.^5^ Briefly, we randomly selected 623 asymptomatic men aged 40–49 years, without clinical CVD or other severe illnesses, residing in Allegheny County, Pennsylvania, USA (n=310 from the voter registration list) or Kusatsu City, Shiga, Japan (n=313 from the Basic Residents’ Register). Recruitment was conducted between 2002 and 2006. All participants gave informed consent. We excluded 53 participants from the present study: 4 participants with missing data for CAC and 49 participants on lipid-lowering medications; we excluded participants taking lipid-lowering medications because lipid-lowering medications could distort the relationship between NMR-measured lipoproteins and CAC. Final sample size was 570 participants: 270 US-White and 300 Japanese.

### Measurement of coronary artery calcium

As described earlier,^5^ EBCT was performed using a GE-Imatron C150 EBCT scanner, GE Medical Systems, South San Francisco, California, USA. From the level of the aortic root to the apex of the heart, scanning was performed using a standardised protocol to obtain 30–40 contiguous 3 mm thick transverse images. All scan data were saved to optical disc. Centrally in the Cardiovascular Institute, the University of Pittsburgh, readings of the scanning were done using a Digital Imaging and Communications in Medicine workstation and software by AccuImage (AccuImage Diagnostic, San Francisco, California, USA). Quantification of CAC was done using a software programme which implements the widely accepted Agatston scoring method. A trained radiology technician who was blinded to each participant’s characteristics and the study centres evaluated the readings. The intrareader reproducibility of non-zero Agatston Coronary Calcium Score (CCS) had an intraclass correlation of 0.99.^5^

### Risk factor assessment

All participants underwent a physical examination, a laboratory assessment and completed a self-administered questionnaire.^5^ We measured body weight and height while the participant was wearing light clothing without shoes and calculated body mass index (BMI) as weight (kg)/height squared (m^2^). Participants were considered smokers if they reported current use of cigarettes or had stopped smoking within the past 30 days. Pack-years of smoking were calculated as years of smoking multiplied by the number of cigarettes per day divided by 20. Those drinking alcohol ≥2 days per week were considered alcohol drinkers.

Venipuncture was performed early in the clinic visit after a 12-hour fast. Serum and plasma samples were stored at −70°C and shipped to the University of Pittsburgh. As mentioned previously,^5^ we assayed glucose, insulin, lipids (including TC, LDL-C, HDL-C and triglycerides), fibrinogen and C-reactive protein (CRP) using serum/plasma samples using standardised methods. We defined diabetes as individuals with fasting glucose ≥7.0 mmol/L or use of medications for diabetes. The participant with systolic blood pressure ≥140 mm Hg and/or diastolic blood pressure ≥90 mm Hg or use of antihypertensive medications was considered as hypertensive.

### Measurement of lipoprotein subclasses

Using serum samples stored at −70°C, NMR spectroscopy (LipoScience, Raleigh, North Carolina, USA) was used to measure lipoprotein subclass particle concentrations and average VLDL, LDL and HDL particle diameters. As the basis for the quantification, the NMR method uses the characteristic signals broadcast by lipoprotein subclasses of different size. The NMR method measured the particle concentrations of the following lipoprotein species: three VLDL subclasses (large, >60 nm; medium, 35–60 nm and small, 27–35 nm), three LDL subclasses (intermediate-density lipoprotein (IDL), 23–27 nm; large, 21.3–23 nm and small, 18.3–21.2 nm) and three HDL subclasses (large, 8.8–13 nm; medium, 8.2–8.8 nm and small, 7.3–8.2 nm). Weighted average particle sizes (average diameters) of VLDL-P, LDL-P and HDL-P were calculated from the subclass levels.

### Statistical analysis

Agatston CCS was categorised into two groups (<10 and ≥10). A CCS cut-off point of 10 was selected due to the possibility that scores ranging from 1 to 9 may be an imaging artefact from a spurious noise and have a low reliability.^17 18^ Sociodemographic characteristics of study participants, traditional lipids, and NMR-measured lipoprotein distributions were analysed per race/ethnicity. Normally distributed continuous variables were expressed as means±SD and compared using a two-sample t-test. Highly skewed continuous variables were expressed as a median and IQR and compared using the Mann-Whitney U test. Categorical variables were expressed in percentages and compared using the χ^2^ statistic.

We used age-adjusted robust Poisson regression for the US-White and the Japanese separately to determine the association of traditional lipids and lipoproteins (particle concentrations and their sizes) with CCS ≥10. We calculated the age-adjusted prevalence ratio (PR) for race/ethnicity (reference group=Japanese) with CCS ≥10 as an outcome. We further adjusted for individual lipids and lipoproteins in separate robust Poisson regression model to assess change in the age-adjusted PR for race/ethnicity. In the age-adjusted model, we also assessed the interaction between race/ethnicity and individual lipoproteins on CAC. To determine the independent effect of race/ethnicity, we used multivariable robust Poisson regression adjusting for traditional cardiovascular risk factors (age, smoking, BMI, hypertension, diabetes, HDL-C, LDL-C and triglycerides), alcohol consumption, inflammatory markers (CRP and fibrinogen) and lipoproteins, which have shown major changes in PR for race/ethnicity in age-adjusted robust Poisson regression models.

For ‘lipoprotein models’ (models with traditional cardiovascular risk factors, alcohol consumption, inflammatory markers and selected lipoproteins), compared with the ‘referent model’ (traditional cardiovascular risk factors, alcohol consumption and inflammatory markers), to assess reclassification improvement by lipoproteins, we used the Net Reclassification Improvement (NRI) index and the Integrated Discrimination Improvement (IDI) index. For NRI, we defined four categories of risk <5.0%, 5.0% to <10.0%, 10.0% to <20.0% and ≥20%. The NRI distinguished the movements in reclassified categories by the observed outcome (CCS ≥10). The IDI measured the mean difference in the predicted probabilities between CCS ≥10 and CCS <10. All p values were two-tailed and p values <0.05 were considered significant. SAS V.9.4 (SAS Institute, Cary, North Carolina, USA) was used for all statistical analyses.

## Results

The prevalence of CCS ≥10 (CAC-prevalence) was 23.0% in US-White and 11.0% in Japanese (table 1). The US-White had higher levels of inflammatory markers (CRP and fibrinogen), fasting insulin and were more obese. The Japanese had less favourable profiles for the presence of hypertension, fasting glucose, smoking status, drinking status and average alcohol consumption per day.

**Table 1:** Demographic and clinical characteristics of US-White men in Allegheny County, USA, and Japanese men in Kusatsu, Japan

Variables	US-White men (n=270)		Japanese men (n=300)		P value
	Mean	SD	Mean	SD	
Age*, years	44.9	2.8	45.1	2.8	0.442
Waist circumference*, cm	98.0	11.3	85.1	8.2	<0.001
Body mass index*, kg/m^2^	27.7	4.0	23.7	3.1	<0.001
Pack-years of smoking**†**	0.0	(0.0–2.0)	18.75	(3.0–29.0)	<0.001
Systolic blood pressure*, mm Hg	122.8	11.4	124.8	15.7	0.092
Hypertension**‡**	13.3	–	25.7	–	<0.001
Current smoker**‡**	7.8	–	49.3	–	<0.001
Current drinker**‡**	45.6	–	67.2	–	<0.001
Alcohol consumption**†,** g/day	4.9	(1.0–16.5)	14.3	(2.4–42.0)	<0.001
Fasting glucose*, mg/dL	101.2	15.4	106.7	18.6	<0.001
Diabetes mellitus**‡**	3.3	–	5.7	–	0.182
Fibrinogen*, µmol/L	8.5	2.1	7.5	1.9	<0.001
Fasting insulin*, µIU/mL	14.7	7.6	10.2	4.4	<0.001
C-reactive protein**†**, nmol/L	8.6	(4.8–17.1)	2.9	(1.9–6.7)	<0.001
Agatston Coronary Calcium Score >10‡	23.0	–	11.0	–	<0.001
Calcium score percentiles (50th, 75th, 90th, 95th)	(0, 8.9, 45.6, 119.8)		(0, 1.5, 13, 37.2)		–

SI conversion factors: to convert glucose to mmol/L, multiply values by 0.0555.

*Continuous normally distributed variables expressed in mean (SD) and compared using a two-sample t-test.

†Continuous non-normally distributed variables expressed in median (IQR) and compared using the Mann-Whitney U test.

‡Categorical variable expressed as percentages and compared using Pearson’s χ^2^ test.

The two populations had similar levels of total LDL-P, small LDL-P, large LDL-P, LDL particle size and total VLDL-P (table 2). The US-White had significantly higher small HDL-P, large VLDL-P and VLDL particle size. The Japanese had significantly higher HDL-C, IDL-P, total HDL-P, medium HDL-P, large HDL-P, HDL particle size and small VLDL-P compared with the US-White.

**Table 2:** Lipoprotein subfractions by chemical analysis and NMR spectroscopy in US-White men in Allegheny County, USA, and Japanese men in Kusatsu, Japan, 2002–2006

Lipids and lipoprotein profile	US-White men (n=270)		Japanese men (n=300)		P value
	Mean	SD	Mean	SD	
**Standard lipids**					
Total cholesterol, mmol/L	5.6	1.0	5.6	0.9	0.554
LDL-C, mmol/L	3.6	0.9	3.4	0.9	0.070
HDL-C, mmol/L	1.2	0.3	1.4	0.4	0.011
Triglycerides, mmol/L	1.7	1.2	1.7	0.9	0.734
**NMR-measured lipoprotein particles**					
*LDL particles*					
Total LDL-P, nmol/L	1480.6	339.6	1469.4	400.9	0.721
Small LDL-P, nmol/L	686.6	358.9	663.1	358.0	0.432
Large LDL-P, nmol/L	661.1	314.1	654.0	254.2	0.774
IDL-P, nmol/L	132.9	100.1	152.3	112.0	0.031
LDL particle size, nm	20.9	0.7	20.9	0.7	0.192
*HDL particles*					
Total HDL-P, μmol/L	31.2	5.8	35.6	6.8	<0.001
Small HDL-P, μmol/L	20.5	4.4	17.2	5.6	<0.001
Medium HDL-P, μmol/L	7.7	4.0	12.1	6.3	<0.001
Large HDL-P, μmol/L	3.1	2.7	6.3	3.6	<0.001
HDL particle size, nm	8.5	0.6	9.1	0.6	<0.001
*VLDL particles*					
Total VLDL-P, nmol/L	83.5	43.2	87.5	47.5	0.285
Small VLDL-P, nmol/L	40.3	24.0	44.9	28.0	0.031
Medium VLDL-P*, nmol/L	32.0	(14.0–53.0)	32.0	(15.0–53.0)	0.813
Large VLDL-P*, nmol/L	3.0	(1.0–7.0)	1.0	(0.0–4.0)	<0.001
VLDL particle size, nm	47.0	7.8	44.8	7.1	<0.001

All continuous nearly normally distributed variables expressed in mean (SD) and compared using a two-sample t-test.

*Continuous variables with skewed distribution were expressed in median (IQR) and compared using the Mann-Whitney U test.

HDL, high-density lipoprotein; IDL, intermediate-density lipoprotein; LDL, low-density lipoprotein;NMR, nuclear magnetic resonance; VLDL, very low-density lipoprotein.

In both populations, total LDL-P and small LDL-P were significantly associated with CCS ≥10. Among US-White, triglycerides, small LDL-P, LDL-P size, IDL, VLDL particle size and large VLDL-P were significantly associated with CCS ≥10 (table 3). Among Japanese, LDL-C, HDL particle size, small HDL-P and large HDL-P were significantly associated with CCS ≥10 (table 3).

**Table 3:** Age-adjusted association (PR (95% CI)) of lipids and lipoproteins subfractions with the CCS ≥10 in US-White men in Allegheny County, USA, and Japanese men in Kusatsu, Japan

Lipids and lipoproteins	US-White men (n=270)	Japanese men (n=300)
**Standard lipids**		
LDL-C	1.23 (0.98 to 1.54)	1.35 (1.04 to 1.76)
HDL-C	0.79 (0.59 to 1.05)	0.94 (0.67 to 1.33)
Triglycerides	1.48 (1.03 to 2.12)	1.28 (0.74 to 2.22)
**NMR-measured lipoprotein particles**		
*LDL particles*		
Total LDL-P	1.34 (1.06 to 1.70)	1.32 (1.04 to 1.69)
Small LDL	1.28 (1.07 to 1.54)	1.29 (1.00 to 1.67)
IDL	1.2743 (1.07 to 1.51)	1.23 (0.95 to 1.58)
Large LDL	0.90 (0.74 to 1.07)	1.01 (0.72 to 1.40)
LDL particle size	0.81 (0.65 to 0.99)	0.94 (0.67 to 1.34)
*HDL particles*		
Total HDL-P	0.94 (0.70 to 1.24)	1.18 (0.88 to 1.56)
Small HDL	0.89 (0.70 to 1.13)	1.39 (1.01 to 1.92)
Medium HDL	1.21 (0.90 to 1.62)	1.08 (0.82 to 1.41)
Large HDL	0.76 (0.54 to 1.08)	0.68 (0.42 to 1.00)
HDL particle size	0.88 (0.68 to 1.12)	0.67 (0.44 to 1.00)
*VLDL particles*		
Total VLDL-P	1.13 (0.93 to 1.36)	1.13 (0.88 to 1.45)
Small VLDL	1.00 (0.77 to 1.29)	1.11 (0.87 to 1.43)
Medium VLDL	1.12 (0.93 to 1.39)	1.24 (0.88 to 1.75)
Large VLDL	1.23 (1.00 to 1.53)	1.15 (0.89 to 1.50)
VLDL particle size	1.26 (1.07 to 1.49)	0.92 (0.69 to 1.24)

Each lipid or lipoprotein is modelled separately, adjusted for age.

CCS, Agatston Coronary Calcium Score; HDL-C, high-density lipoprotein cholesterol; HDL-P, high-density lipoprotein particles; IDL, intermediate-density lipoprotein; LDL-C, low-density lipoprotein cholesterol; LDL-P, low-density lipoprotein particles;NMR, nuclear magnetic resonance; PR, prevalence ratio; VLDL-P, very low-density lipoprotein particles.

Table 4 describes the percent change in the age-adjusted PR for CCS ≥10 in the US-White compared with the Japanese. The age-adjusted prevalence of CCS ≥10 for the US-White was 2.14 times higher compared with the Japanese. Major changes (>5%) in the PR of CCS ≥10 for US-White were seen with further adjustment for HDL-C, HDL-P size, VLDL particle size, medium HDL-P, large HDL-P and large VLDL-P. In a model including age and large HDL-P, the prevalence of CCS ≥10 for the US-White was 1.62 times higher compared with Japanese. There was ~25% reduction in the age-adjusted PR for the US-White after adjustment for large HDL-P. There was no significant interaction between lipids or lipoproteins and CCS ≥10 by race/ethnicity.

**Table 4:** Per cent change in the age-adjusted PR for CCS ≥10 in the US-White men with the addition of lipids/lipoproteins

Robust Poisson regression models	Race/Ethnicity	Per cent change in PR*	Lipids and lipoproteins
	PR (95% CI)		PR (95% CI)
Age	2.14 (1.46 to 3.13)	–	–
**Age and standard lipids**			
Age+LDL-C	2.12 (1.45 to 4.00)	–0.90	1.28 (1.08 to 1.51)
Age+HDL-C	1.99 (1.34 to 2.94)	–7.00	0.85 (0.68 to 1.06)
Age+triglycerides	2.17 (1.48 to 3.17)	–0.47	1.41 (1.05 to 1.92)
**Age and NMR-measured lipoprotein particles**			
*Age and LDL particles*			
Age+total LDL-P	2.19 (1.49 to 3.20)	+2.34	1.33 (1.13 to 1.58)
Age+small LDL-P	2.10 (1.44 to 3.08)	–1.87	1.29 (1.08 to 1.49)
Age+large LDL-P	2.13 (1.45 to 3.12)	–0.47	0.91 (0.77 to 1.08)
Age+IDL-P	2.22 (1.51 to 3.25)	+3.74	1.25 (1.08 to 1.45)
Age+LDL particle size	2.17 (1.48 to 3.18)	+1.40	0.84 (0.71 to 1.01)
*Age and HDL particles*			
Age+total HDL-P	2.18 (1.47 to 3.23)	+1.87	1.03 (0.84 to 1.26)
Age+small HDL-P	2.04 (1.34 to 3.10)	–4.67	1.08 (0.89 to 1.32)
**Age+medium HDL-P**	**2.35 (1.54 to 3.59**)	**+9.81**	1.12 (0.93 to 1.37)
**Age+large HDL-P**	**1.62 (1.03 to 2.54**)	–**24.30**	0.72 (0.54 to 0.95)
**Age+HDL particle size**	**1.73 (1.09 to 2.74**)	–**19.16**	0.80 (0.63 to 1.00)
*Age and VLDL particles*			
Age+total VLDL-P	2.16 (1.48 to 3.16)	+0.90	1.13 (0.97 to 1.31)
Age+small VLDL-P	2.17 (1.47 to 3.16)	+1.40	1.04 (0.87 to 1.25)
Age+medium VLDL-P**†**	2.15 (1.47 to 3.14)	+0.47	1.15 (0.95 to 1.39)
**Age+large VLDL-P†**	**2.00 (1.32 to 2.90**)	–**6.54**	1.19 (1.01 to 1.42)
**Age+VLDL particle size**	**2.00 (1.36 to 2.92**)	–**6.54**	1.16 (1.00 to 1.34)

Bold font indicates a major change (>5%) in PR for race/ethnicity.

Each lipid or lipoprotein was modelled separately in a model adjusted for age.

Per cent change in PR for race compared with the age-adjusted model.

*Per cent change in PR compared with age-adjusted model was calculated as: (*(PR for race/ethnicity in the age-adjusted model)–(PR for race in a given respective model))×100/(PR for race/ethnicity in an age-adjusted model)*.

†Medium VLDL-P and large VLDL-P numbers were log-transformed after the addition of one unit. All other continuous variables were standardised.

CCS, Agatston Coronary Calcium Score; HDL-C, high-density lipoprotein cholesterol; HDL-P, high-density lipoprotein particles; IDL-P, intermediate-density lipoprotein particles; LDL-C, low-density lipoprotein cholesterol; LDL-P, low-density lipoprotein particles; PR, prevalence ratio; VLDL-P, very low-density lipoprotein particles.

The US-White were 2.03 times more likely to have CCS ≥10 compared with Japanese after adjustment for traditional cardiovascular risk factors (table 5). With further adjustment for alcohol consumption and inflammatory markers, the PR of CCS ≥10 for US-White increased nearly to 2.10. Major changes in the PR of CCS ≥10 for US-White were noticed with further adjustment for large HDL-P or VLDL-P size or large VLDL-P. Major attenuation (15%–8%) in the PR of CCS ≥10 for US-White was seen after including large HDL-P and VLDL-P/VLDL particle size (models III-A/III-B) together in a model including CVD risk factors. In a fully adjusted model (model IV-A) including CVD risk factors, total LDL-P, large HDL-P and VLDL particle size, US-White had 1.73 times greater prevalence of CCS ≥10 compared with Japanese men. Based on NRI and IDI, no significant reclassification improvement was found with the addition of total LDL-P, large HDL-P and VLDL-P/ VLDL particle size to the referent model (table 5).

**Table 5:** Multivariable-adjusted PR of race/ethnicity, % change in PR, IDI and NRI for CCS ≥10 when NMR-measured lipoproteins were added to referent model

Robust Poisson regression models	PR (95% CI)	% change in PR compared with model II*	IDI (*SE*)	NRI (*SE*)
Model I	2.03 (1.23 to 3.34)	–	–	–
Model II**†**	2.10 (1.24 to 3.48)	–	–	–
Model II-A	1.86 (1.07 to 3.26)	−11.43	0.000 (0.002)	0.028 (0.025)
Model II-B	2.08 (1.18 to 3.47)	−1.00	0.000 (0.000)	0.006 (0.005)
Model II-C	1.98 (1.18 to 3.31)	−5.71	0.003 (0.002)	−0.004 (0.025)
Model II-D	2.01 (1.26 to 3.47)	−4.30	0.000 (0.000)	0.006 (0.011)
Model III-A	1.75 (1.05 to 3.08)	−16.67	0.003 (0.003)	−0.006 (0.031)
Model III-B	1.80 (1.07 to 3.24)	−14.29	0.000 (0.002)	0.026 (0.025)
Model IV-A	1.73 (1.02 to 3.08)	−17.62	0.003 (0.003)	−0.021 (0.033)
Model IV-B	1.79 (1.07 to 3.23)	−14.76	0.001 (0.002)	0.028 (0.022)

Model I: race, age, BMI, pack-year of smoking, hypertension, diabetes, triglyceride, LDL-C, HDL-C.

Model II-A: model II+large HDL-P.

Model II-B: model II+HDL particle size.

Model II-C: model II+VLDL particle size.

Model II-D: model II+large VLDL-P.

Model III-A: model II+large HDL-P+VLDL particle size.

Model III-B: model II+large HDL-P+large VLDL-P.

Model IV-A: model II+total LDL-P+large HDL-P+VLDL particle size.

Model IV-B: model II+total LDL-P+large HDL-P+large VLDL-P.

Reclassification categories for NRI: <5.0%, 5.0%–9.9%, 10.0%–19.9% and high ≥20%.

CRP, triglycerides, ethanol intake, large VLDL-P and medium VLDL-P were log transformed after addition of one unit. All other continuous variables were standardised.

*Per cent change in PR for race compared with model II was calculated as: *((PR for race/ethnicity in model II)–(PR for race/ethnicity in each model (model II-A to IV-B)))×100/(PR for race/ethnicity in model II)*.

†Model II (*referent model*): model I+alcohol intake+CRP+fibrinogen.

CRP, C reactive protein; HDL-C, high-density lipoprotein cholesterol; HDL-P, high-density lipoprotein particles; IDI, Integrated Discrimination Improvement;LDL-C, low-density lipoprotein cholesterol; LDL-P, low-density lipoprotein particles; NRI, Net Reclassification Improvement; PR, prevalence ratio; VLDL-P, very low-density lipoprotein particles.

## Discussion

In a community-based sample of healthy middle-aged men, the US-White had 2.10 times higher prevalence of CCS ≥10 compared with the Japanese adjusting for traditional cardiovascular risk factors, alcohol consumption and inflammatory markers. This higher prevalence of CCS ≥10 in the US-White was partially attenuated with further adjustment for large HDL-P and large VLDL-P/VLDL particle size. Previous studies have reported an inverse association of large HDL-P^19^ and positive association of large VLDL-P and larger VLDL particle size with atherosclerosis and CVD.^10^ In our study, the US-White compared with the Japanese had significantly lower large HDL-P, higher large VLDL-P and larger VLDL particle size, which could explain the partial attenuation in the prevalence ratio of having CCS ≥10 among US-White after adjustment for large HDL-P and higher large VLDL-P or larger VLDL particle size.

The finding of significantly higher large HDL-P and larger HDL particle size among Japanese compared with the US-White is compatible with previous findings mentioned on differences between the two populations in certain lifestyle factors such as alcohol consumption, eating of fish and lean BMI. Increased alcohol consumption, higher fish intake and lean BMI are reported to be directly associated with the activity of cholesterol ester transfer protein (CETP),^20^ which is associated with higher total HDL-P, large size HDL particles and a higher concentration of total HDL-C^20-22^. Since, the Japanese eat more fish, drink more alcohol and have lower BMI than their US counterparts, as shown in this study^17^ and other studies,^21 22^ these specific features of the Japanese men may have increased their HDL particle size, large HDL-P concentration as well as total HDL-C.

The finding of larger VLDL particle size and higher large VLDL-P in the US-White compared with the Japanese is consistent with reports from the literature on plasma concentrations of lipoproteins and obesity.^23^ The prevalence of obesity is likely to be substantially increased in the US-White compared with the Japanese during the time of the survey and in the past,^24^ and therefore the lifetime burden of (exposure to) obesity differs in these two populations. One potential mechanism for an association between obesity and atherosclerosis is increased plasma concentrations of triglyceride-rich large VLDL-P and larger VLDL particle size.^25^ Triglyceride-rich large VLDL-P are found in the human intima and have been isolated from atherosclerotic lesions. Large VLDL-P has high-affinity for LDL receptors and binds to unique triglyceride-rich lipoproteins/apoB48 receptors expressed specifically on monocytes, macrophages and endothelial cells.^26^ Large VLDL-P cause rapid, receptor-mediated macrophage lipid accumulation and are related to the progression of atherosclerosis in humans.^27^ Increased secretion of large VLDL-P also favours the transfer of triglyceride from triglyceride-rich lipoprotein to LDL-P and HDL-P through the action of CETP.^28^ Subsequently, increased hepatic lipase activity converts the triglyceride-rich LDL-P and HDL-P to small LDL-P and small HDL-P, respectively.^28^ Furthermore, this process is also related to the lowered HDL-C which was noted to have a relatively dose-response relationship to reduced levels of large HDL-P. Consistent with the lipoprotein metabolism theory mentioned above, in our study, the US-White had higher small HDL-P and lower total HDL-P, large HDL-P and HDL-C compared with the Japanese.

Differences in CAC-prevalence because of lifetime exposure to traditional risk factors is unlikely because available data from national or population-based surveys indicate that the US-White and the Japanese have had very similar levels of TC and blood pressure from childhood to adulthood.^3^ Furthermore, the US-White have much lower rates of cigarette smoking than the Japanese.^3^ Differences in CAC-prevalence between the US-White and the Japanese because of genetic factors is very unlikely because a study of Japanese migrants to the USA showed that the Japanese residing in America (Japanese-Americans) have similar or higher levels of subclinical atherosclerosis compared with the US-White.^5^ In a supplementary analysis, the Japanese-Americans compared with Japanese residing in Japan had significantly different NMR-measured lipoprotein particle distributions. Japanese-Americans had a significantly higher prevalence of CCS ≥10 compared with the Japanese after adjustment for CHD risk factors (PR=2.83; 95% CI=1.67 to 4.78), and this difference was partially attenuated (12%–18%) with further adjustment of large HDL-P and large VLDL-P (online supplementary tables 1 and 2).

10.1136/openhrt-2019-001119.supp1 Supplementary data

One plausible mechanism for the difference in the prevalence of CAC between the two populations is increased insulin resistance among US-White indicated by higher BMI. Insulin resistance is independently associated with the CAC-prevalence in the US-White^29^ and the Japanese.^30^ The US-White are expected to be more insulin resistant because they had significantly higher BMI compared with the Japanese. Their lipid and lipoprotein profile (higher large VLDL-P, small HDL-P, larger VLDL particle size and lower total HDL-P, medium HDL-P, large HDL-P, small VLDL-P and smaller HDL particle size) is also consistent with the lipid and lipoprotein profile seen in insulin-resistant individuals.^23^ However, in this study with further adjustment for fasting insulin or homeostasis model assessment of insulin resistance (HOMA-IR) (insulin (IU/L)×(glucose (mg/dL))/22.5) above other CHD risk factors, attenuation in the difference in the CAC-prevalence between the two populations was very minimal (data not shown). Also, the magnitude of reduction in the difference in the CAC-prevalence between two populations after adjusting for large HDL-P and large VLDL-P/VLDL particle size above CHD risk factors was very similar with or without adjustment for fasting insulin or HOMA-IR (data not shown).

Findings of the present study should be considered in light of important limitations. First, the study is cross-sectional in design, and we cannot confirm any causality between CAC and lipoprotein. Second, our study examined apparently healthy men aged 40–49 years in the USA or Japan only, therefore, the results of the study cannot be generalised to other populations and age groups. Third, despite CAC being a reliable biomarker of coronary atherosclerosis and independently predicts CHD,^1^ CAC may not detect some atherosclerosis plaques, because it is not a direct measure of coronary atherosclerosis. Fourth, we analysed blood samples obtained at a one-time point only. Fifth, although we adjusted for several covariates in multivariable logistic regression, we cannot exclude the possibility of residual confounding because of unmeasured variables.

This study has several strengths: first, this was the first community-based study trying to explore the association of NMR lipoprotein distributions and difference in the CAC-prevalence between the US-White and the Japanese; second, all laboratory analyses and CAC measurements were conducted at the same laboratory; third, the age range of the studied population was narrow 40–49 years possibly providing greater precision for examining the stated hypothesis in an apparently healthy population. We focused on male gender and age group 40–49 years because population levels of TC and blood pressure have been similar in these US-White and Japanese populations throughout their lifetime.^3^

## Conclusion

In a community-based sample of asymptomatic US-White and Japanese men aged 40–49 years, the US-White had significantly different lipoprotein particle distributions compared with the Japanese. Despite having an adverse profile for major independent cardiovascular risk factors among Japanese, the US-White had a significantly higher CAC-prevalence compared with the Japanese, and this CAC-prevalence difference could not be entirely attributed to variations in the distribution of lipoproteins. Our findings support the notion that there is a common source of exposure in the diet among the Japanese, which may account for their lower rates of atherosclerosis and CHD.^3^ Further investigations are needed.
