# Supplementary material for: Lipoprotein particles and coronary artery calcium in middle-aged US-White and Japanese men
Source: Open Heart. 2019 Dec 29;6(2):e001119. doi: 10.1136/openhrt-2019-001119 (PMC6937418; doi:10.1136/openhrt-2019-001119)
Supplement: Supplementary data [file openhrt-2019-001119supp001.pdf]

Supplementary Tables

Supplementary table 1. Age-adjusted prevalence ratio for CCS ≥10 in Japanese-American men in Honolulu, US compared to Japanese men in Kusatsu, Japan (Reference group for race/ethnicity = Japanese)

**Robust Poisson Regression Models**	**Race/Ethnicity** PR (95% CI)	**Percent change in PR[a]**	**Lipids and lipoproteins** PR (95% CI)
Age	2.30 (1.56, 3.40)	-	-
	Age and Standard Lipids		
Age + LDL-C	2.41 (1.64, 3.53)	+4.78	1.24 (1.05, 1.46)
Age + HDL-C	2.25 (1.52, 3.33)	-2.17	1.00 (0.84, 1.19)
Age + Triglycerides	2.25 (1.52, 3.32)	-2.17	1.31 (1.00, 1.72)
	Age and NMR-measured Lipoprotein Particles		
Age and LDL particles			
Age + Total LDL-P	2.45 (1.68, 3.59)	+6.52	1.21 (1.13, 1.31)
Age + Small LDL-P	2.18 (1.47, 3.21)	-5.22	1.24 (1.14, 1.34)
Age + Large LDL-P	2.17 (1.42, 3.32)	-5.65	0.94 (0.77, 1.15)
Age + IDL-P	2.35 (1.59, 3.47)	+2.17	1.16 (1.00, 1.36)
Age + LDL Size	2.24 (1.51, 3.32)	-2.61	0.95 (0.79, 1.13)
Age and HDL particles			
Age + Total HDL-P	2.25 (1.52, 3.33)	-2.17	1.14 (0.94, 1.39)
Age + Small HDL-P	1.86 (1.21, 2.87)	- 19.13	1.31 (1.04, 1.65)
Age + Med HDL-P	2.32 (1.57, 3.43)	+0.87	1.05 (0.88, 1.27)
Age + Large HDL-P	2.06 (1.37, 3.10)	- 10.43	0.82 (0.66, 1.02)
Age + HDL Size	2.20 (1.45, 3.32)	- 4.35	0.96 (0.80, 1.15)
Age and VLDL particles			
Age + Total VLDL-P	2.23 (1.51, 3.30)	-3.04	1.13 (0.98, 1.31)
Age + Small VLDL-P	2.29 (1.55, 3.38)	-0.40	1.10 (0.95, 1.27)
Age + Med VLDL-P[b]	2.20 (1.49, 3.26)	-4.35	1.22 (1.00, 1.50)
Age +Large VLDL-P[b]	2.15 (1.43, 3.23)	- 6.52	1.10 (0.94, 1.29)
Age + VLDL Size	2.27 (1.53, 3.36)	- 1.30	0.98 (0.84, 1.16)

PR, Prevalence ratio; Each lipid or lipoprotein was modeled separately in a model adjusted for age;
[a] Percent change in PR for race compared to age-adjusted model,
[a] Percent change in PR compared to age-adjusted model was calculated as: *[(PR for race in an age-adjusted model) – (PR for race in a given respective model)]*100 / [PR for race in an age-adjusted model]*
Bold font in above table indicates major change in PR for race
[b] Variables Medium VLDL-P and large VLDL-P were log transformed after addition of one unit. All other continuous variables were standardized.

Supplementary table 2. Multivariable-adjusted PR of race/ethnicity, change in PR, IDI, and NRI for CCS ≥10 when NMR-measured lipoproteins were added to referent model

**Robust Poisson Regression Models**	**PR (95% CI)**	**% change in PR compared to model II[a]**	**IDI (*p*-value)**	**NRI (*p*-value)**
Model I	2.74 (1.67, 4.51)	-	-	-
Model II[b]	2.83 (1.67, 4.78)	-	-	-
Model II-A	2.42 (1.46, 4.02)	-14.49	0.008 (0.18)	0.064 (0.15)
Model II-B	2.76 (1.66, 4.59)	-2.47	0.000 (0.97)	0.015 (0.26)
Model II-C	2.79 (1.72, 4.55)	+1.41	0.003 (0.46)	0.077 (0.02)
Model II-D	2.74 (1.66, 4.53)	-3.18	0.001 (0.33)	0.014 (0.41)
Model III-A	2.50 (1.54, 4.07)	-11.66	0.012 (0.07)	0.056 (0.24)
Model III-B	2.48 (1.49, 4.12)	-12.37	0.014 (0.04)	0.071 (0.13)
Model IV-A	2.39 (1.44, 3.97)	-15.55	0.013 (0.06)	0.070 (0.14)
Model IV-B	2.33 (1.38, 3.94)	-17.67	0.016 (0.02)	0.111 (0.03)

PR, Prevalence ratio; IDI, Integrated Discrimination Improvement; NRI, Net Reclassification Improvement; Reclassification categories for NRI: <5.0%, 5.0-9.9%, 10.0-19.9%, and high ≥20%;
Model I: race, age, BMI, pack -year of smoking, hypertension, diabetes, triglyceride, LDL-C, HDL-C
[b]Model II (*Referent Model*): model I + alcohol intake + CRP + fibrinogen
Model II-A: model II + large HDL-P
Model II-B: model II + HDL particle size
Model II-C: model II + VLDL particle size
Model II-D: model II + large VLDL-P
Model III-A: model II + large HDL-P + VLDL size
Model III-B: model II + large HDL-P + large VLDL-P
Model IV-A: model II + total LDL-P + large HDL-P + VLDL size
Model IV-B: model II + total LDL-P + large HDL-P + large VLDL-P
[a]Percent change in PR for race compared to model II was calculated as: *[(PR for race/ethnicity in model II) – (PR for race/ethnicity in each model (model II-A to IV-B))]*100/ [PR for race/ethnicity in model II]*
